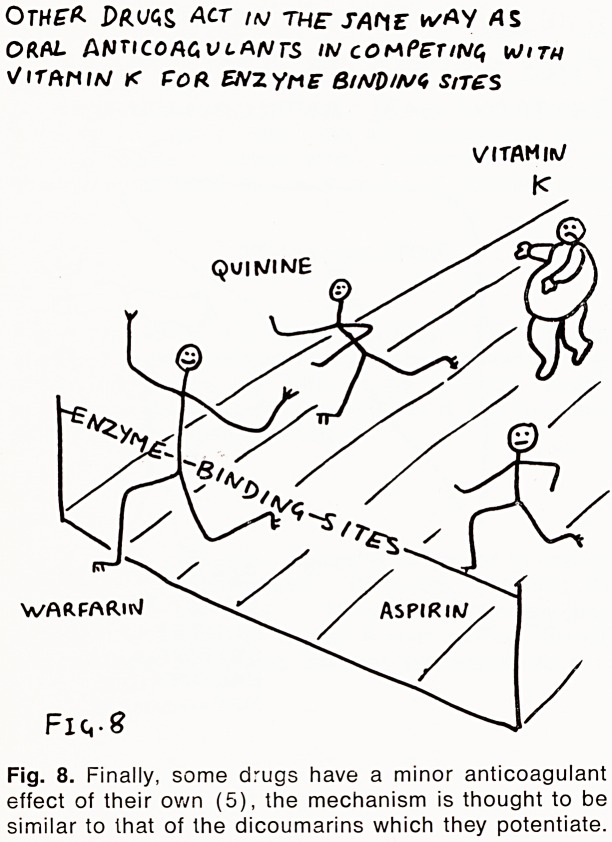# The Problems of Anticoagulant Control—1. Drug Interactions

**Published:** 1970-10

**Authors:** T. J. Hamblin

**Affiliations:** from the Department of Pathology and University Department of Medicine, Southmead Hospital


### The Problems of Anticoagulant Control

### 1. DRUG INTERACTIONS

**T. J. Hamblin, M.B., Ch.B.**
from the Department of Pathology and University Department of Medicine,
Southmead Hospital

The potentially lethal effects of oral anticoagulants call for careful monitoring of the state of the clotting mechanism. It is because of this close scrutiny that so much is known of the factors influencing their action.

Oral anticoagulants act by competitive inhibition of vitamin K, which is essential for the production of certain clotting factors: notably Factors VII, IX, and X, and Prothrombin. Vitamin K is therefore the physiological antagonist of oral anticoagulants, and when given in pharmacological doses eventually reverses their effect. However, variations of vitamin K in the diet (it is found in dark green vegetables) will lead to fluctuation in anticoagulant control. More commonly the difficulty in achieving stable anticoagulation is associated not with dietary idiosyncracy, but with the interaction of other drugs. The following case history illustrates this point: (See Fig. 1) — Mr. R. was anticoagulated with Warfarin following a deep venous thrombosis and a pulmonary embolus. His control was at first satisfactory on 8 mg. daily, but he continued to have pain in the affected leg. To alleviate this, he was given a week's course of phenylbutazone; at the end of the week he complained of haematuria. His prothrombin ratio at this stage was 7.0, although his Warfarin dosage had remained unaltered. All drug therapy was stopped, and when the bleeding had ceased Warfarin was restarted at a lower dose. Control was re-established at 6mg. daily, but after a further two weeks, the dose had to be increased. Despite successive increases it was not possible to reach a satisfactory level of anticoagulation. He then revealed that following the frightening symptom of haematuria he had needed a barbituate sleeping tablet every night. When this was replaced by Nitrazepam he became well controlled on Warfarin 8 mg. daily.

FIG. 1.

The list of drugs either potentiating or antagonising the oral anticoagulants is a long one. Remembering lists is tedious, and once remembered one list is very like another. Many people remember things visually; for them the following cartoons may prove as useful as they have been for me.

**Fig. 2.** Whether or not the vitamin K produced by intestinal flora is absorbed is in dispute. However, the killing of normal gut bacteria is thought to be the most likely reason why broad spectrum antibiotics potentiate anticoagulants (3). There is no dispute about liquid paraffin, which has an unpredictable effect on anticoagulant control, since it interferes with the absorption of both vitamin K and oral anticoagulants (7).

**Fig. 3.** Vitamin K is a fat soluble vitamin, and is only absorbed in the presence of bile salts. Cholestyramine, which renders the bile salts in the intestine unavailable, decreases absorption of vitamin K and therefore potentiates oral anticoagulants (6).

**Fig. 4.** In the circulation vitamin K is carried in association with the triglycerides. Clofibrate, (Atromid S) which lowers the level of plasma triglycerides, also reduces the amount of vitamin K that can be carried, and therefore potentiates the oral anticoagulants (9).

FIG. 2.

FIG. 3

FIG. 4.

THE PROTEIN BINDING LADDER.

FIG. 5

THE ENZYME INDUCERS STOKE THE FIRES AND FAN THE FLAMES THAT DESTROY ORAL ANTICOAGULANTS

FIG. 6.

THE ENZYME INHIBITORS TEND TO PUT THE FIRE OUT.

FIG. 7.

**Fig. 5.** In common with many acidic drugs anticoagulants are bound to plasma proteins; only the small unbound fraction being active. The number of such binding sites is limited and drugs that are preferentially bound will displace drugs with a less firm grip on the rungs of this protein binding ladder. It should be noted that not only are oral anticoagulants potentiated by a number of drugs (2), (4), (9) and (10), they themselves are able to displace drugs (4), and thus enhance the side effects of long acting suphonamides, the hypoglycaemia of the sulphonylureas, and the marrow toxicity of methotrexate.

**Fig. 6.** Anticoagulants are metabolised by liver enzymes. Many drugs are capable of non-specific induction of these enzymes (1) and (8), and when this occurs anticoagulants are consumed more rapidly and thus exert less of an effect.

**Fig. 7.** On the other hand, some drugs inhibit these same enzymes (11) and (12), so that consumption of and anticoagulant is delayed and its effect potentiated.

**Fig. 8.** Finally, some drugs have a minor anticoagulant effect of their own (5), the mechanism is thought to be similar to that of the dicoumarins which they potentiate.

### REFERENCES

1. **Corn, M.** (1966), Effect of Phenobarbital and Glutethemide on Biological Half-life of Warfarin. Thrombosis et Diathesis Haemorrhagica (Stuttgart) **16,** 606.

2. **Eissen, M. J.** (1964). Combined Effect of Sodium Warfarin and Phenylbutazone. Journal of the American Medical Association, **189,** 64.

3. **Koller, F.** (1958). Side Effects and Contraindications of Anticoagulants Thrombosis et Diathesis. Haemorrhagica (Stuttgart) 2, 604.

4. **McIver, A. K.** (1967). Drug Interactions. The Pharmaceutical Journal. **197,** 205.

5. **Meyer, L. and Herxheimer, A.** (1968). Side Effects of Drugs. Excerpta Medica Foundation, Amsterdam, Vol. VI, p.466.

6. **Mishkell, M. A.** (1968). Xanthomatosis, British Journal of Hospital Medicine. **1,** 223.

7. **Moore, C. B.** (1964). Anticoagulant Therapy, Angiology. **15,** 27.

8. **Oddessky, L., Weiss, M., Dayton, P. G.** (1966). The Effect of Chloral Hydrate on Bishydroxycoumarin Metabolism. Journal of the American Medical Association. **197,** 366.

9. **Oliver, M. F., Roberts, S. D., Hayes, D., Partridge, J. F., Suzman, M. M., Bersohn, I.** (1963). Effect of Atromid and Ethylchlorphenoxyisobutyrate on Anticoagulant Requirements. Lancet. **i,** 143.

10. **Olwin, J. A., Arscott, P. M., Koppel, J. L.** (1958). Choice and Control of Anticoagulant Drugs. Geriatrics. **13,** 773.

11. **Pyörälä, K., Kekki, M.** (1963). Decreased Anticoagulant tolerance during Methandrostenolene therapy. Scandinavian Journal of Clinical and Laboratory Investigation. **15,** 367.

12. **Weiner, M.** (1966). Effect of Centrally Active Drugs on the Action of Coumarin Anticoagulants. Nature (London). **212,** 1599.

## Figures and Tables

**FIG. 1. f1:**
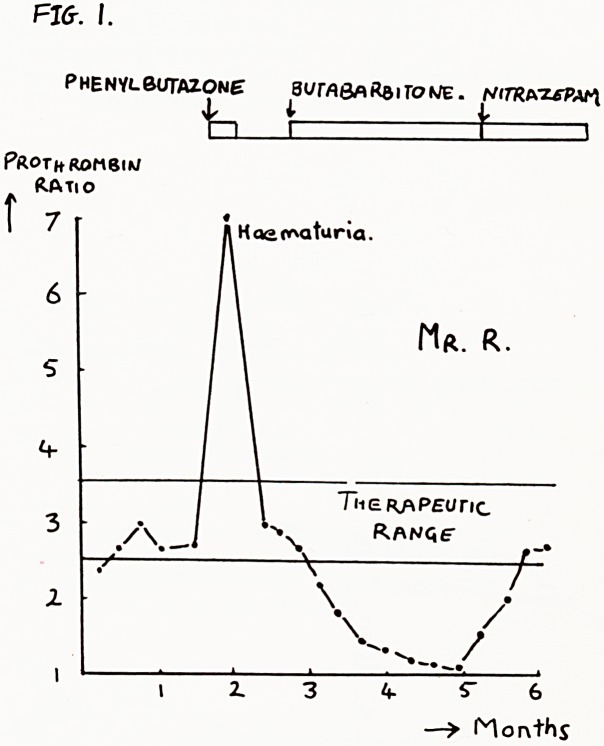


**Fig. 2. Fig. 3. Fig. 4. f2:**
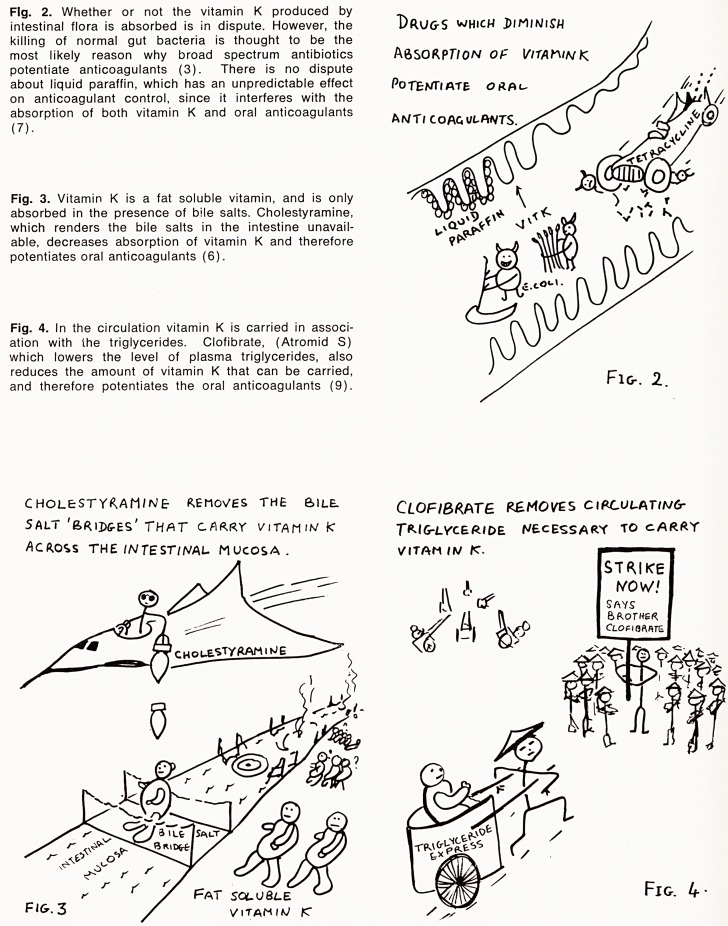


**Fig. 5. Fig. 6. Fig. 7. f3:**
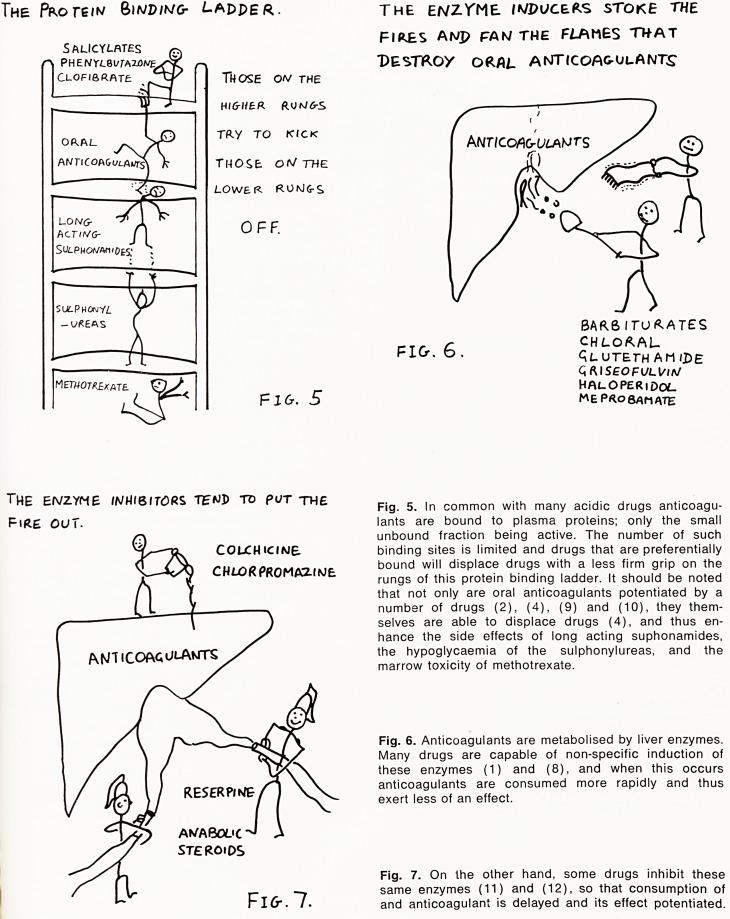


**Fig. 8. f4:**